# Draft Genome Sequence of *Rhodococcus* sp. Strain 311R

**DOI:** 10.1128/genomeA.00378-15

**Published:** 2015-05-21

**Authors:** Elham Ehsani, Ruy Jauregui, Robert Geffers, Michael Jareck, Nico Boon, Dietmar H. Pieper, Ramiro Vilchez-Vargas

**Affiliations:** a University of Ghent, Ghent, Belgium; b Microbial Interactions and Processes Research Group, Helmholtz Centre for Infection Research, Braunschweig, Germany; c Genome Analytics, Helmholtz Centre for Infection Research, Braunschweig, Germany

## Abstract

Here, we report the draft genome sequence of *Rhodococcus* sp. strain 311R, which was isolated from a site contaminated with alkanes and aromatic compounds. Strain 311R shares 90% of the genome of *Rhodococcus erythropolis* SK121, which is the closest related bacteria.

## GENOME ANNOUNCEMENT

Members of the genus *Rhodococcus* are aerobic, Gram-positive, nonmotile, nonsporulating, slow-growing, high-GC, and nocardioform actinomycetes (1). *Rhodococcus* species show remarkable metabolic versatility, including the ability to degrade hexane (2), benzene (3), polychlorinated biphenyl (4), polybrominated diphenyl ethers (5), and aromatic alcohols (6), and they are able to bioconvert a diverse range of organic compounds into triacylglycerols for use as biofuels (7). Moreover, *Rhodococcus equi* has been described as a pathogen responsible for bronchopneumonia in young foals (8, 9), and recently, *Rhodococcus* sp. strain BG43, closely related to *Rhodococcus erythropolis*, has been described as a degrader of the *Pseudomonas* quinolone signal, a quorum-sensing signal molecule employed by the opportunistic pathogen *Pseudomonas aeruginosa* (10).

Here, we present the draft genome sequence of *Rhodococcus* sp. strain 311R (taxon identification [ID] 1617904), isolated from soil of a hydrocarbon-contaminated environment (11–13) and capable of growing in benzene, decane, phenol, or anthranilate as a sole carbon source.

The genome was sequenced using the Illumina MiSeq platform, which generated paired-end reads sequences of 250 bp, and assembled using Edena (14, 15), producing 128 contigs with a total genome size of 6,343,721 bp (62.57% G+C content; *N*_50_, 88.31 Kbp; mean, 49.22 Kb), with an average of 43.7-fold coverage. Automatic annotation was performed using the RAST server version 4.0 (16), generating 6,091 features potentially assigned to protein-coding genes (open reading frames [ORFs]).

A comparison between the draft genome of 311R and the 12 genomes/draft genomes of *Rhodococcus* sp. DK17 (17), *Rhodococcus* sp. JVH1 (18), *R. jostii* RHA1 (19), *R. erythropolis* PR4 (20), *R. erythropolis* SK121 (BioProject PRJNA55853), *R. erythropolis* CCM2595 (21), *R. erythropolis* R138 (22), *R. opacus* B4 (3), *R. opacus* PD630 (7), *R. equi* 103S (8), *R. pyridinivorans* SB3094 (23), and *Rhodococcus* sp. Chr-9 (24) showed that the closest strain to 331R is *R. erythropolis* SK121, with an average 90.5% (amino acid sequence) ORF similarity. The two strains share 5,445 ORFs, with >80% similarity (88% of the whole genome), and 354 ORFs observed in the genome of the strain 311R are absent from the genome of strain SK121, indicating that these strains belong to different species.

### Nucleotide sequence accession number.

This draft genome sequencing project has been deposited in the European Nucleotide Archive under the accession number CFHW00000000.
